# Interventions for the Management of Bladder Spasms in Adults with Indwelling Urinary Catheters: A Nursing Practice-Oriented Systematic Review

**DOI:** 10.17533/udea.iee.v44n1e13

**Published:** 2026-03-31

**Authors:** Gayatri Padval, Jasneet Kaur

**Affiliations:** 1 Ph.D. Scholar. Email: catchgayatri@gmail.com https://orcid.org/0009-0009-6114-9013 Symbiosis International University India catchgayatri@gmail.com; 3 Department of Nursing, Symbiosis College of Nursing, Symbiosis International (DeemedUniversity), Pune, India Symbiosis International University Department of Nursing Symbiosis College of Nursing Symbiosis International (DeemedUniversity) Pune India

**Keywords:** Bladder spasm, Catheter-related bladder discomfort, Indwelling urinary catheter, Nursing interventions, vejiga urinaria, espasmo, catéteres urinarios, atención de enfermería., bexiga urinária, espasmo, cateteres urinários, cuidados de enfermagem.

## Abstract

**Objective.:**

To critically synthesize evidence on pharmacological and non-pharmacological interventions relevant to nursing practice for the prevention and management of bladder spasms and catheter-related bladder discomfort in adults with indwelling urinary catheters.

**Methods.:**

A systematic review was conducted in accordance with the Preferred Reporting Items for Systematic Reviews and Meta-Analyses (PRISMA) guidelines. Electronic databases including PubMed, CINAHL, MEDLINE, and Scopus were searched for English-language studies published from 2015 to 2025. Randomized controlled trials, quasi-experimental, and prospective studies involving adult patients with indwelling urinary catheters were included.

**Results.:**

Ten studies met the inclusion criteria. Both pharmacological and non-pharmacological interventions were effective in reducing bladder spasms and catheter-related bladder discomfort. Non-pharmacological, nursing-led interventions-such as catheter balloon volume adjustment, neuromodulatory techniques, evidence-based targeted nursing care, structured nursing management models, and Traditional Chinese Medicine-based nursing approaches-demonstrated consistent reductions in symptom severity and improvements in patient comfort. Pharmacological therapies, including vitamin C, solifenacin, and ketamine, were primarily effective for short-term symptom relief. Overall, four studies were assessed as having low risk of bias, five as moderate risk, and one as high risk.

**Conclusion.:**

Nursing-led, non-pharmacological interventions play a central role in managing bladder spasms in patients with indwelling urinary catheters and should be prioritized in clinical practice. Further high-quality studies with standardized outcome measures are required to strengthen the evidence base.

## Resumen

**Objetivo.:**

Sintetizar de forma crítica la evidencia sobre las intervenciones farmacológicas y no farmacológicas relevantes para la práctica de la enfermería en la prevención y el tratamiento de los espasmos vesicales y las molestias vesicales relacionadas con el catéter en adultos con catéteres urinarios permanentes.

**Métodos.:**

Se realizó una revisión sistemática de acuerdo con las directrices Preferred Reporting Items for Systematic Reviews and Meta-Analyses (PRISMA). Se realizaron búsquedas en bases de datos electrónicas, como PubMed, CINAHL, MEDLINE y Scopus, para encontrar estudios publicados en inglés de 2015 a 2025. Se incluyeron ensayos controlados aleatorios, estudios cuasi-experimentales y prospectivos en los que participaron pacientes adultos con catéteres urinarios permanentes. La extracción de datos y la evaluación de la calidad metodológica se realizaron utilizando herramientas estandarizadas adecuadas al diseño del estudio.

**Resultados.:**

Diez estudios cumplieron los criterios de inclusión. Tanto las intervenciones farmacológicas como las no farmacológicas fueron eficaces para reducir los espasmos vesicales y las molestias vesicales relacionadas con el catéter. Las intervenciones no farmacológicas dirigidas por el personal de enfermería, como el ajuste del volumen del globo del catéter, las técnicas neuromoduladoras, los cuidados de enfermería específicos basados en la evidencia, los modelos estructurados de gestión de enfermería y los enfoques de enfermería basados en la medicina tradicional china, demostraron reducciones consistentes en la gravedad de los síntomas y mejoras en la comodidad de los pacientes. Las terapias farmacológicas, como la vitamina C, la solifenacina y la ketamina, fueron eficaces principalmente para el alivio de los síntomas a corto plazo. En general, se evaluó que cuatro estudios tenían un riesgo bajo de sesgo, cinco un riesgo moderado y uno un riesgo alto.

**Conclusión.:**

Las intervenciones no farmacológicas dirigidas por el personal de enfermería desempeñan un papel fundamental en el tratamiento de los espasmos vesicales en pacientes con catéteres urinarios permanentes y deben ser prioritarias en la práctica clínica. Se necesitan más estudios de alta calidad con medidas de resultados estandarizadas para reforzar la base empírica.

## Resumo

**Objetivo.:**

. Sintetizar criticamente as evidências sobre intervenções farmacológicas e não farmacológicas relevantes para a prática de enfermagem na prevenção e tratamento de espasmos da bexiga e desconforto urinário relacionado com cateteres em adultos com cateteres urinários de demora.

**Métodos.:**

Foi realizada uma revisão sistemática de acordo com as diretrizes Preferred Reporting Items for Systematic Reviews and Meta-Analyses (PRISMA). Foram pesquisadas bases de dados eletrônicas, incluindo PubMed, CINAHL, MEDLINE e Scopus, para estudos em inglês publicados de 2015 a 2025. Foram incluídos ensaios clínicos randomizados, estudos quase experimentais e prospectivos envolvendo pacientes adultos com cateteres urinários de demora. A extração de dados e a avaliação da qualidade metodológica foram realizadas utilizando ferramentas padronizadas adequadas ao desenho do estudo.

**Resultados.:**

Dez estudos atenderam aos critérios de inclusão. Tanto as intervenções farmacológicas quanto as não farmacológicas foram eficazes na redução dos espasmos da bexiga e do desconforto urinário relacionado ao cateter. Intervenções não farmacológicas lideradas por enfermeiros - como ajuste do volume do balão do cateter, técnicas neuromoduladoras, cuidados de enfermagem direcionados baseados em evidências, modelos estruturados de gestão de enfermagem e abordagens de enfermagem baseadas na medicina tradicional chinesa - demonstraram reduções consistentes na gravidade dos sintomas e melhorias no conforto do paciente. Terapias farmacológicas, incluindo vitamina C, solifenacina e cetamina, foram eficazes principalmente para o alívio dos sintomas a curto prazo. No geral, quatro estudos foram avaliados como tendo baixo risco de viés, cinco como risco moderado e um como alto risco.

**Conclusão.:**

As intervenções não farmacológicas conduzidas por enfermeiros desempenham um papel central no tratamento dos espasmos da bexiga em pacientes com cateteres urinários de demora e devem ser priorizadas na prática clínica. São necessários mais estudos de alta qualidade com medidas de resultados padronizadas para fortalecer a base de evidências.

## Introduction

Indwelling urinary catheters are among the most frequently used medical devices in acute, perioperative, and long-term care settings.^1^ Although they play a vital role in urinary drainage, fluid monitoring, and postoperative management, their use is associated with a wide range of infectious and non-infectious complications.^2^ Beyond catheter-associated urinary tract infections, patients commonly experience catheter-related bladder discomfort (CRBD), urine leakage, blockage, and painful bladder spasms, all of which significantly affect comfort, recovery, and quality of life.^3^ Bladder spasms result from involuntary detrusor muscle contractions, often triggered by mechanical irritation of the bladder wall, catheter balloon pressure, traction on the catheter, or inflammation.^4^ Clinically, bladder spasms may manifest as suprapubic pain, urgency, a strong desire to void despite catheterization, and leakage around the catheter. These symptoms are distressing for patients and can lead to agitation, sleep disturbance, reduced mobility, and dissatisfaction with care.^5^ In postoperative and long-term catheterized patients, bladder spasms are also associated with secondary complications such as urethral trauma, catheter bypassing, and prolonged hospitalization.^6^

From a nursing perspective, bladder spasms represent a critical yet often under-recognized aspect of catheter-related care.^7^ Nurses are primarily responsible for catheter insertion assistance, fixation, maintenance, monitoring of urine output, early identification of complications, and implementation of comfort-promoting interventions.^8^ Daily nursing decisions-such as catheter securement, positioning, drainage bag management, bowel care, patient education, and timely assessment of discomfort-directly influence the occurrence and severity of bladder spasms. When catheter removal is not clinically feasible, nursing-led interventions become essential in minimizing patient distress and preventing further complications.^9^ Pharmacological treatments, including antimuscarinic agents and other medications, have been used to manage bladder spasms; however, these are often associated with adverse effects such as dry mouth, constipation, blurred vision, and sedation, which may be particularly problematic in older adults and postoperative patients.^10^ Consequently, there is growing interest in non-pharmacological and nursing-driven strategies, such as catheter balloon volume adjustment, catheter fixation techniques, transcutaneous electrical stimulation, acupressure, traditional nursing methods, and evidence-based targeted nursing care.^11^ These interventions are generally low-cost, non-invasive, and feasible within routine nursing practice.

Despite the clinical relevance of bladder spasms, guidance for nurses on effective prevention and management strategies remains fragmented.^12^ Existing studies vary widely in design, population, interventions, and outcome measures, making it difficult to translate evidence into consistent nursing practice. Furthermore, many reviews focus predominantly on medical or anaesthetic interventions, with limited emphasis on the nursing role in assessment, prevention, and management of bladder spasms in patients with indwelling urinary catheters.^13^

Therefore, a systematic synthesis of the available evidence from a nursing perspective is warranted. This systematic review aims to critically examine and summarize pharmacological and non-pharmacological interventions relevant to nursing practice for the management of bladder spasms in adults with indwelling urinary catheters. By consolidating current evidence, this review seeks to support nurses in delivering evidence-based, patient-centered catheter care and to identify gaps for future nursing research. This systematic review was guided by the following research questions: (1) What pharmacological and non-pharmacological interventions are effective in preventing or managing bladder spasms and catheter-related bladder discomfort in adults with indwelling urinary catheters? (2) Which nursing-led and non-pharmacological interventions demonstrate the greatest effectiveness in reducing the incidence, frequency, and severity of bladder spasms and improving patient comfort? And (3) What are the clinical implications of the identified interventions for nursing practice in the management of patients with indwelling urinary catheters?

## Methods

This systematic review was conducted to synthesize existing evidence on nursing-relevant pharmacological and non-pharmacological interventions for the management of bladder spasms in adults with indwelling urinary catheters. To ensure methodological rigor, transparency, and reproducibility, this review was conducted in accordance with the Preferred Reporting Items for Systematic Reviews and Meta-Analyses (PRISMA) guidelines.

**Eligibility criteria. *Inclusion criteria*.** Studies were eligible for inclusion if they met the following criteria: (i) included adult participants aged 18 years and above with an indwelling urinary catheter; (ii) involved medical, surgical, or postoperative patients with a catheter in situ; (iii) examined pharmacological and/or non-pharmacological interventions relevant to nursing practice for the prevention or management of bladder spasms or catheter-related bladder discomfort; (iv) reported outcomes related to bladder spasms, pain, discomfort, frequency, or severity; (v) used experimental, quasi-experimental, or randomized controlled trial designs; (vi) were published in the English language; and (vii) were published within the 2015 to 2025. ***Exclusion criteria.*** Studies were excluded if they: (i) involved pediatric populations; (ii) focused on patients with neurological or neurogenic bladder disorders; (iii) editorials, commentaries, opinions, or book chapters; (iv) lacked full-text availability; or (v) did not report outcomes related to bladder spasms or catheter-related bladder discomfort.

PICO Framework. The review question was formulated using the Population, Intervention, Comparison, and Outcome (PICO) framework. The population included adult patients aged 18 years and older with indwelling urinary catheters in medical, surgical, or postoperative settings. The interventions comprised pharmacological and non-pharmacological strategies relevant to nursing practice for the prevention or management of bladder spasms and catheter-related bladder discomfort, including catheter management techniques, neuromodulatory interventions, structured nursing care models, and medication therapy. Comparators included routine catheter care, standard postoperative care, placebo, or alternative interventions where applicable. Outcomes of interest included the incidence, frequency, severity, and duration of bladder spasms, catheter-related bladder discomfort, pain scores, patient comfort, and satisfaction.

Search strategy. A comprehensive search strategy was developed to identify relevant studies addressing bladder spasms in patients with indwelling urinary catheters. Electronic databases searched included PubMed, CINAHL, MEDLINE, and Scopus. Boolean operators (AND/OR) were applied to combine search terms and refine results. Searches were conducted using a combination of Medical Subject Headings (MeSH) and free-text terms. Key search terms included ((((((((Urinary Bladder) AND (spasm)) AND (Urinary Catheters)) OR (Catheters, Indwelling)) OR (Catheters)) OR (Catheterization)) AND (Nursing Care)) AND (Pain Management)) AND (Patient Comfort)

Study Selection and Screening Process. The study selection process followed the Preferred Reporting Items for Systematic Reviews and Meta-Analyses (PRISMA) guidelines. All records identified through database searching were exported into a reference management system, and duplicate studies were removed prior to screening. Titles and abstracts were independently screened to assess eligibility based on the predefined inclusion and exclusion criteria. Studies that were clearly irrelevant were excluded at this stage. Full-text articles of the remaining studies were then retrieved and assessed for eligibility. Any studies that did not meet the inclusion criteria or failed to report outcomes related to bladder spasms or catheter-related bladder discomfort were excluded after full-text review. Discrepancies during the screening and selection process were resolved through discussion and consensus. The selection process resulted in a total of 10 studies being included in the final review. A PRISMA flow diagram was used to illustrate the identification, screening, eligibility, and inclusion of studies.

Data Extraction. Data extraction was performed systematically to ensure consistency and accuracy across the included studies. A standardized data extraction form was developed by the reviewers prior to data collection. The following information was extracted from each eligible study: author(s) and year of publication, study design, sample size and participant characteristics, clinical setting, type of indwelling urinary catheter, details of the intervention and comparator, duration of intervention, outcome measures related to bladder spasms or catheter-related bladder discomfort, and key findings. Both pharmacological and non-pharmacological interventions relevant to nursing practice were included. Outcomes of interest primarily focused on the incidence, frequency, severity, and duration of bladder spasms, as well as pain scores, patient comfort, and satisfaction where reported. Data were extracted independently and cross-checked to minimize errors and ensure completeness. Any discrepancies in extracted data were resolved through discussion and consensus.

Quality Assessment and Risk of Bias. The methodological quality of the included studies was assessed using the Cochrane Risk of Bias Tool for randomized controlled trials and the Joanna Briggs Institute Critical Appraisal Checklists for non-randomized studies. Of the ten included studies, four were classified as having a low risk of bias, which demonstrated clear intervention protocols, validated outcome measures, and adequate reporting.^14^^-^^17^ Five studies were assessed as having a moderate risk of bias due to limitations such as lack of blinding, single-center design, or incomplete reporting of allocation concealment.^18^^-^^21^ These assessments were considered during data synthesis and interpretation.

**Figure1:**
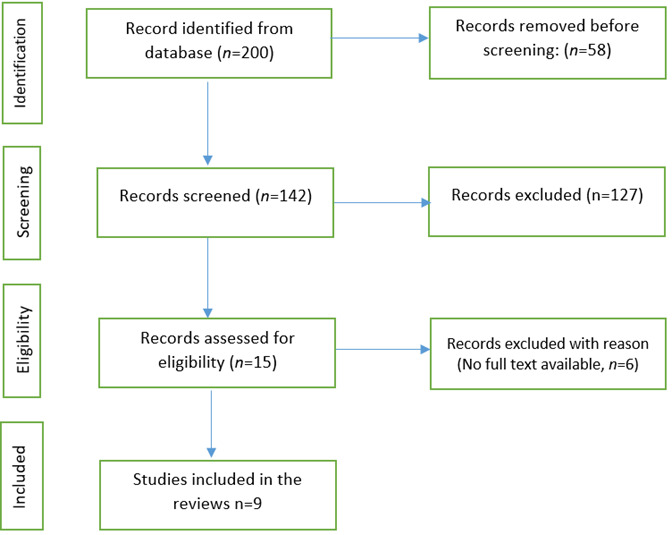
PRISMA flow chart

**Table 1:** Characteristics of Included studies

Author	Design	Sample size	Intervention	Key findings
Zugail, *et al*. 2019.^17^	Non randomized control - prospective study.	49 samples	Catheter balloon volume reduced by 50%; patients served as their own controls with assessments conducted before and 2 hours after intervention. Visual Analogue Scale (VAS) for pain and Catheter-Related Bladder Discomfort (CRBD) questionnaire.	Reduction of catheter balloon volume by 50% significantly decreased pain scores (VAS 2.80 to 2.02) and CRBD severity (1.02 to 0.75) (*p* < 0.05), indicating improved catheter tolerance.
Chanjuan Zhang, Zhiying Xiao. 2017^16^	Prospective random controlled design.	66 samples randomly divided into two groups: the control group n = 36 and the Experimental group n = 30 who underwent prostate or bladder surgeries.	The control group received the routine postoperative care. The experimental group received daily transcutaneous electrical stimulation of the foot during 3 days after surgery; each time lasted for 60 min. All patients were evaluated by the Visual Analogue Scale for pain sensation, frequency of bladder spasm episodes, and a total score of bladder spasms symptoms.	Pain scores, bladder spasm frequency, and symptom severity decreased in both groups over 3 days (*p* < 0.001); however, the intervention group demonstrated significantly lower VAS pain and symptom scores on postoperative days 2 and 3 compared with controls (*p* < 0.05).
Qiao Tian, Xichen Zhang, Meixia Luo, *et al*. 2023.^21^	RCT	99 samples.50 in control & 49 in experimental group.	A quality control circle-based standardized nursing management model was implemented for continuous bladder irrigation, with pre- and post-intervention comparisons of bladder spasm incidence, catheter obstruction, and patient and nurse satisfaction.	Quality control circle intervention markedly reduced bladder spasms and catheter obstruction and improved patient and nurse satisfaction.
Juan Yu*et al.* 2024.^22^	RCT	104 samples having Experimental group & control group.	Traditional Chinese Medicine (TCM). Nursing method such as guidance for anxiety, adjusting catheter position etc.	The application of traditional Chinese medicine therapy for bladder spasm is cost-effective, efficient, and easy to implement.
Xiaoying Liu *et al*. 2020.^19^	RCT	Random sample of 72 patients who underwent electrovaporisation of prostate divided into experimental & control group.	Evidence-based targeted nursing care delivered by a multidisciplinary urology team, including staff training, individualized preoperative patient education, psychological support, catheter fixation, functional rehabilitation exercises, and use of antispasmodic medication when indicated.	Targeted nursing care significantly reduced the incidence, frequency, duration, and pain severity of bladder spasms compared with standard care (*p* < 0.05).
Farahani *et al.* 2024.^18^	Clinical trial with a randomized control group, which was performed as a single-blind, single-centre study.	160 patients with LUTS/BPH were assigned to suprapubic catheterization using Pezzer (*n* = 80) or three-way Foley catheters (*n* = 80).	Interview with the patient and physical examination to evaluate evidence related to painful bladder spasm, complete Bladder Spasm Symptom Scale questionnaire / checklist and the visual analogue scale (VAS).	Foley catheterization resulted in significantly lower bladder spasm severity and pain compared with Pezzer catheterization following open prostatectomy.
Jun Young Park *et al*. 2023.^15^	A double blind randomized placebo controlled study.	Experimental & control group (*n* = 59 respectively).	Vitamin C 1gm IV given to experimental group. Control group received normal saline, after the induction of anesthesia.	Experimetal group exihibited significantly lower incidence of moderate or greater CRBD immediate postoperative and improved patients satisfaction following TUR of bladder.
Liang *et al*. 2021.^14^	RCT	70 Male patients undergoing TURP under GA.	8 Acupressure points applied to the patients through self adhesive cutaneous electrode pads with acupoint stimulator, for 30 min. with frequency of 2/100 HZ.	The findings of the study indicate that Trasncutaneous electrical Acupoint stimulation (TEAS)could significantly prevent incidence and severity of CRBD and having benefit of noninvasive and nonpharmacological Modality.
Xu Feng Peng *et al.* 2017.^20^	RCT	Experimental group 165 & control group 150 samples who underwent urethroplasty.	Patient in experimental group was treated with solifenacin for 7 days placebo was given to control group. VAS was used to measure severity of bladder spasm.	The short term therapy with solifenacin is an effective and safe method for decreasing the frequency of bladder spasm after urethroplasty.

## Results

### Study Selection

The database search identified a total of 200 records. After removal of 58 duplicate records, 142 studies remained for title and abstract screening. Of these, 127 studies were excluded for not meeting the inclusion criteria. Fifteen full-text articles were assessed for eligibility, and five were excluded due to irrelevant outcomes or study populations. Finally, 9 studies met the inclusion criteria and were included in this systematic review.

### Characteristics of Included Studies

The ten included studies were published between 2015 and 2025 and involved adult patients aged 18 years and older with indwelling urinary catheters in postoperative or clinical settings. Study designs included randomized controlled trials, prospective non-randomized studies, and quasi-experimental studies. Sample sizes ranged from 49 to 315 participants. Most studies were conducted in surgical populations, particularly patients undergoing transurethral resection of the prostate,^14^^,^^16^^,^^22^ bladder surgery,^15^ urethroplasty,^20^ or open prostatectomy,^18^ while other studies focused on postoperative catheter management following prostate surgery using targeted nursing or catheter-related interventions.^17^^,^^19^^,^^21^

### Intervention Types

The included studies evaluated both non-pharmacological and pharmacological interventions for the management of bladder spasms or catheter-related bladder discomfort. Non-pharmacological interventions included reduction of catheter balloon volume,^17^ transcutaneous electrical stimulation of the foot,^16^ transcutaneous electrical acupoint stimulation,^14^ Traditional Chinese Medicine-based nursing methods,^22^ evidence-based targeted nursing care,^19^ quality control circle nursing management models,^21^ and catheter type modification.^18^ Pharmacological interventions included the administration of vitamin C,^15^ and solifenacin.^20^

### Effects of Non-Pharmacological Interventions

Non-pharmacological interventions consistently demonstrated reductions in the incidence, frequency, and severity of bladder spasms. Reduction of catheter balloon volume resulted in significantly lower pain and discomfort scores.^17^ Transcutaneous electrical stimulation of the foot and transcutaneous electrical acupoint stimulation significantly reduced postoperative bladder spasm severity and catheter-related bladder discomfort.^14^^,^^16^ Targeted nursing interventions and quality control circle-based nursing management models were associated with a marked decrease in bladder spasm incidence, reduced catheter obstruction, and improved patient satisfaction.^19^^,^^21^ Traditional Chinese Medicine nursing approaches also showed beneficial effects in reducing bladder spasm symptoms and were reported to be cost-effective and easy to implement.^22^

### Effects of Pharmacological Interventions

Pharmacological interventions demonstrated varying degrees of effectiveness. Intravenous vitamin C significantly reduced the incidence of moderate to severe catheter-related bladder discomfort in the immediate postoperative period, although the effect was not sustained beyond six hours.^15^ Solifenacin therapy effectively reduced the frequency and severity of bladder spasms following urethroplasty, with minimal adverse effects reported.^20^

### Outcome Measures

Across studies, outcomes were measured using validated tools such as the Visual Analogue Scale, catheter-related bladder discomfort symptom scores, frequency and duration of bladder spasms, analgesic requirements, and patient satisfaction.^14^^-^^17^^,^^20^ Overall, most interventions resulted in statistically significant improvements in bladder spasm-related outcomes compared with standard care.^19^^,^^21^^,^^22^

## Discussion

This systematic review critically examined pharmacological and non-pharmacological interventions relevant to nursing practice for the management of bladder spasms and catheter-related bladder discomfort (CRBD) in adults with indwelling urinary catheters. Overall, the findings indicate that bladder spasms are a multifactorial complication influenced by mechanical, physiological, and psychosocial factors, and that nursing-led and non-pharmacological interventions play a crucial role in reducing symptom severity and improving patient comfort.^17^^,^^19^^,^^21^^,^^22^

Non-pharmacological interventions demonstrated consistent and clinically meaningful benefits across multiple studies. Reduction of catheter balloon volume was shown to significantly decrease pain and discomfort scores, supporting the hypothesis that mechanical irritation of the bladder wall and trigone is a major contributor to CRBD.^17^ This finding aligns with earlier evidence suggesting that excessive balloon inflation increases detrusor over activity and bladder neck irritation.^23^ From a nursing perspective, balloon volume adjustment represents a simple, low-cost, and immediately actionable intervention that can be implemented safely when clinically appropriate.^24^

Neuromodulation-based approaches, including transcutaneous electrical stimulation of the foot and transcutaneous electrical acupoint stimulation (TEAS), were also effective in reducing the incidence and severity of bladder spasm.^14^^,^^16^ These findings are consistent with previous systematic reviews indicating that afferent nerve modulation can inhibit detrusor overactivity and improve bladder control.^25^ The non-invasive nature of these interventions makes them particularly attractive for nursing implementation, especially in postoperative settings where pharmacological options may be limited due to side effects or contraindications.^26^

Targeted nursing interventions and structured nursing management models emerged as particularly impactful in reducing bladder spasm incidence and improving patient satisfaction. Evidence-based targeted nursing care and quality control circle-based management models were associated with fewer spasms, reduced catheter obstruction, and enhanced patient comfort.^19^^,^^21^ These findings reinforce the growing body of literature emphasizing that catheter-related complications are strongly influenced by nursing practices such as catheter fixation, positioning, drainage bag management, bowel care, and patient education.^27^ Importantly, these interventions address not only physiological mechanisms but also modifiable care processes, highlighting the central role of nursing vigilance and clinical judgment.

Traditional Chinese Medicine-based nursing methods also showed promising results in reducing bladder spasm symptom.^22^ While these approaches are supported by plausible neuromodulatory mechanisms, their integration into mainstream practice remains limited by variability in protocols and cultural applicability. Nevertheless, similar complementary approaches have been reported to improve postoperative comfort in urological patients,^28^ suggesting a potential role as adjunctive therapies when delivered within evidence-informed frameworks.

Pharmacological interventions, including vitamin C, solifenacin, and ketamine, were effective primarily in the short-term reduction of CRBD. Vitamin C significantly reduced moderate to severe CRBD in the immediate postoperative period, although the effect was transient.^15^ Antimuscarinic therapy with solifenacin effectively reduced bladder spasm frequency with minimal adverse effects, consistent with prior randomized trials.^20^^,^^29^ However, the routine use of pharmacological agents is limited by side effects, particularly in older adults and patients with comorbidities, reinforcing the importance of prioritizing non-pharmacological and nursing-led strategies whenever possible. 

Despite these positive findings, several limitations within the evidence base warrant consideration. Many studies were single-center with small sample sizes and limited blinding, contributing to moderate risk of bias in a substantial proportion of the included studies.^17^^,^^19^^,^^21^^,^^22^ Additionally, heterogeneity in outcome measures and follow-up duration limits direct comparison across interventions.^14^^-^^16^ These methodological limitations have also been highlighted in previous reviews of CRBD management,^3^ underscoring the need for standardized outcome measures and longer-term follow-up.

From a clinical and nursing perspective, the findings of this review support a multimodal, patient-centered approach to bladder spasm management. Mechanical optimization of catheter care, structured nursing protocols, neuromodulatory techniques, and selective pharmacological use should be integrated based on individual patient needs and clinical context.^30^ Importantly, empowering nurses through education, protocol development, and decision-making authority may significantly reduce catheter-related complications and improve patient experience.

### Clinical Implications for Nursing Practice

The findings of this systematic review highlight the critical role of nurses in the prevention and management of bladder spasms and catheter-related bladder discomfort in patients with indwelling urinary catheters. Nurses are uniquely positioned to identify early signs of bladder spasms through ongoing patient assessment, pain evaluation, and monitoring of catheter function, allowing for timely intervention before symptoms escalate.^31^ Routine nursing practices such as appropriate catheter fixation, avoidance of unnecessary traction, maintenance of unobstructed urine flow, and regular bowel assessment to prevent constipation are essential in minimizing mechanical irritation and detrusor overactivity.^32^

Non-pharmacological interventions demonstrated strong clinical relevance and feasibility within nursing practice. Adjusting catheter balloon volume, implementing structured nursing care protocols, and applying neuromodulatory techniques such as transcutaneous electrical stimulation or acupoint stimulation can significantly reduce bladder spasm severity without exposing patients to medication-related adverse effects.^14^^,^^16^^,^^17^ These interventions are cost-effective, minimally invasive, and can be incorporated into routine postoperative and long-term catheter care with appropriate training and institutional support. The review also underscores the importance of evidence-based nursing models, including targeted nursing care and quality control circle approaches, in reducing bladder spasm incidence and improving patient satisfaction.^19^^,^^21^ Such models emphasize interdisciplinary collaboration, standardized care pathways, and continuous quality improvement, reinforcing the need for nurse-led initiatives in catheter management. Pharmacological interventions should be considered as adjuncts rather than first-line strategies, particularly in older adults and patients at risk of anticholinergic side effects.^20^ Overall, integrating structured assessment, mechanical optimization of catheter care, patient education, and selective use of non-pharmacological and pharmacological interventions can enhance patient comfort, reduce catheter-related complications, and improve quality of care. Ongoing education and empowerment of nurses are essential to translating evidence into consistent clinical practice.

### Strengths and Limitations

This systematic review employed a comprehensive search across multiple databases and included a range of study designs, allowing for a broad evaluation of pharmacological and non-pharmacological interventions relevant to nursing practice. The use of standardized quality appraisal tools strengthened the methodological rigor, and the focus on nursing-led and non-pharmacological strategies enhances the clinical relevance of the findings. However, the review is limited by heterogeneity among included studies in terms of interventions, outcome measures, and follow-up duration, which restricted direct comparison and prevented meta-analysis. Several studies demonstrated moderate risk of bias due to small sample sizes, lack of blinding, and single-center designs. In addition, the inclusion of only English-language publications may have introduced language bias. These limitations underscore the need for well-designed, multicenter randomized trials with standardized outcome measures.

## Conclusion

This systematic review highlights that bladder spasms and catheter-related bladder discomfort are common and distressing complications in adults with indwelling urinary catheters, with significant implications for patient comfort and quality of care. The evidence demonstrates that non-pharmacological, nursing-led interventions-such as optimization of catheter balloon volume, structured nursing care models, neuromodulatory techniques, and appropriate catheter management-are effective, feasible, and associated with fewer adverse effects compared with pharmacological approaches. Pharmacological therapies, including antimuscarinic agents and adjunctive medications, may be beneficial for short-term symptom control but should be used selectively due to potential side effects.

The findings underscore the central role of nurses in the prevention, early identification, and management of bladder spasms through evidence-based catheter care, patient education, and individualized interventions. Despite promising results, variability in study design and outcome measures limits definitive conclusions regarding the superiority of specific interventions. Future research should focus on high-quality, multicenter randomized controlled trials with standardized outcome measures and longer follow-up periods to strengthen the evidence base. Integrating evidence-based nursing interventions into routine catheter care has the potential to improve patient comfort, reduce catheter-related complications, and enhance overall quality of care.
